# The Implications of Policies on the Welfare of Free-Roaming Cats in New Zealand

**DOI:** 10.3390/ani12030237

**Published:** 2022-01-19

**Authors:** Christine L. Sumner, Jessica K. Walker, Arnja R. Dale

**Affiliations:** Royal New Zealand Society for the Prevention of Cruelty to Animals (SPCA NZ), Auckland 0640, New Zealand; christine.sumner@spca.nz (C.L.S.); jessica.walker@spca.nz (J.K.W.)

**Keywords:** companion cat, stray cat, feral cat, cat legislation, human-animal bond

## Abstract

**Simple Summary:**

Free-roaming cats in New Zealand include companion, stray, and feral cats, mostly defined based on their relationship with people. As such, the different policy mechanisms in New Zealand related to addressing the impacts of free-roaming cats reflect these different types of relationships. In this paper, we review the current laws and related regulations, codes, plans, and local bylaws related to cat management and identify the implications they have on cat welfare. Currently, there is no national law for cat management in New Zealand; however, we suggest that there is reason to create national legislation to ensure that cat management is humane and consistent across New Zealand.

**Abstract:**

A lack of national legislation for cat management in New Zealand poses challenges for ensuring that practices are consistently humane and effective. In this paper, we review the current cat management policies in New Zealand and the implications they have on the welfare of free-roaming cats (from here on, referred to as ‘cats’). Our review demonstrates that there are multiple policy mechanisms used to manage cats in New Zealand for a variety of reasons, including animal welfare, pest management, and nuisance, and that these different policies have both positive and negative implications for cat welfare. We provide context pertaining to New Zealanders’ acceptance of current or future laws and regulations and compare the New Zealand policy landscape with other countries, with a particular emphasis on Australia, to identify potential directions and outcomes of increased regulation. We discuss the future of the regulatory environment in New Zealand, including the need to better understand the impact of policies on cats, people, and other animals in urban, rural, and wild spaces. We further discuss the need to better understand the cat–human relationship for future policy decisions and offer a solution based on national cat legislation.

## 1. Introduction

The welfare of free-roaming cats in New Zealand and elsewhere is impacted by human interactions, including those driven by laws and regulations. Free-roaming cats in New Zealand include companion, stray, and feral cats (with approximately 11% of companion cats kept from roaming) [1]. These three categories of cats are contingent on the human–cat relationship, with, at one extreme, companion cats being entirely dependent on humans to meet their needs and, at the other end, feral cats, by definition, having none of their needs met by humans, with stray cats having many of their needs indirectly supplied by humans [2]. As such, the laws and regulations in New Zealand related to the different types of cats reflect the relational differences between the categories of cats and humans.

In this paper, we review the current cat management laws and regulations in New Zealand and the implications these policies have on the welfare of free-roaming cats (from here on, referred to ‘cats’). Many of these policies have implications for other outcomes, such as protecting wildlife and pastoral animals, and reducing nuisance. However, our focus here is cat welfare. We then discuss New Zealanders’ acceptance of current or future regulations and compare the New Zealand regulatory landscape with other countries, with a particular emphasis on Australia, to identify potential directions and outcomes of increased regulation. It is not our intention to provide an in-depth review of other countries’ legislations and, where appropriate, we point the reader to more in-depth treatment of the topic.

We discuss the future of the regulatory environment in New Zealand, including the need to better understand the impact of policies on cats, people, and other animals in urban, rural, and wild spaces. We further discuss the need to better understand the cat–human relationship for future regulations and offer a solution based on national cat legislation.

## 2. Review

We have organised this paper according to the increasingly closer degree of the relationship between cats and people. Free-roaming cats are part of a metapopulation, where companion, stray, and feral cat populations overlap and influence each other [3,4]. Therefore, including laws and regulations that impact all three types of cats is instructive for understanding how they impact free-roaming cat welfare. When a welfare impact is included, this was based on a consensus among the authors or a stated purpose of legislation.

### 2.1. Feral Cat Management

In New Zealand, feral cats are defined as having no relationship with humans, and do not inhabit areas near humans [2]. Feral cats are considered an important predator of wildlife in New Zealand [5,6], and much of the attention these cats receive is based on their negative impacts on native wildlife and biodiversity.

#### 2.1.1. Laws and Regulations for Controlling Feral Cats

The laws in New Zealand that impact feral cat welfare fall into two broad categories: those that enable the control of feral cats as pests, and those that protect a feral cat from unnecessary suffering. Under the Wildlife Act 1953, feral cats are not protected wildlife [7]. The Biosecurity Act 1993 allows the control of feral cats as pests in a pest management plan [8]. There is currently no national pest management plan for feral cats; however, they are included in nearly all regional pest management plans in New Zealand. Under the Conservation Act 1987, feral cats are managed as pests on public conservation land [9]. The Animal Welfare Act 1999 does not make it unlawful to hunt or kill a feral cat “in a wild state” or as a “wild animal or pest” in accordance with provisions of the laws previously listed [10]. (The Animal Welfare Act 1999 also does not prohibit the killing of stray or companion cats if performed humanely.) Feral cats are provided some protections under the Animal Welfare Act, 1999, in that it is an offense to commit wilful or reckless ill treatment towards them, and that if live trapped, there are established periods of time within which the trap must be checked once set, and that when found in a trap, the animal must be removed, attended to properly, or killed without delay [10].

#### 2.1.2. Attitudes towards Feral Cats

The separation of feral cats from other categories of cats within New Zealand has been suggested as a demonstration of reduced empathy for feral cats as they are designated a pest [11]. There is some evidence that the more likely a person is to consider a cat a pest, the less likely they are concerned with their welfare [12], which has implications for the acceptability for how feral cat management is conducted. The impact of feral cats on wildlife is well documented [13,14] and generally accepted by the public. At least one study in New Zealand found that members of the public were mostly concerned about impacts from feral cats, unmanaged strays, and colony cats on native and non-native wildlife compared to companion cats [15].

Additionally, that feral cats are independent of humans to the extent that they are considered a wild animal may impact welfare considerations. Concerns for their welfare are mostly focused on humanely killing them, which often comes down to the method used (e.g., shooting, trapping, poisoning), rather than the actual act of killing [16,17]. Non-lethal control methods for feral cats are preferred over lethal control methods by the public; however, inadequate justification and use of lethal control may be poorly supported by the public [12].

#### 2.1.3. Comparison of Laws and Regulations for Feral Cats

Feral cats are managed in Australia under the threat abatement plan for predation by feral cats under the Environment Protection and Biodiversity Act 1999 (EPBC) [18]. Feral cats are nationally declared pests and environment ministers must remove identified barriers to their effective and humane control. At the state and local government level, there are varying pieces of legislation that relate to feral cats which permit their control as pests [19]. For a more in-depth review of this topic, please see [19].

Attitudes towards feral cats in Australia are similar to those in New Zealand, where feral cats are widely considered a pest, but where values for their companion and stray counterparts have some influence on how members of the public view feral cat management [17]. Recent research has found consistent views among Australians for the support in controlling feral cats and protecting native wildlife [20]. People who engage in feral cat control do so out of concern for native wildlife, because feral cats are considered pests, or it is routine practice [21]. Most survey respondents agreed that feral cats are detrimental to native wildlife, with fewer agreeing that they posed additional harms to humans and livestock [21]. Those survey respondents that did not support feral cat control preferred non-lethal control, such as trap–neuter–return (TNR), and felt that other methods were inhumane, ineffective, and did not address other threats to biodiversity [21]. Other recent research found that gender and familiarity with a method of control influenced acceptability (e.g., cage trapping and shooting, baiting with poisons); women with less familiarity with a method were less likely to find lethal control acceptable compared to men with more familiarity with a method [22].

### 2.2. Stray Cat Management

Unowned, semi-owned, and lost or abandoned companion cats can all be considered stray cats in New Zealand. The degree of socialisation with humans varies from full socialisation to none, with the distinction in this category that these cats are dependent on humans either directly or indirectly. There are no specific laws or regulations related to TNR and, given that this topic has had considerable attention paid to it, we discuss it further later in this article.

#### 2.2.1. Laws and Regulations for Controlling Stray Cats

There are few laws or regulations specific to stray cats. Some of these sources of legislation and regulation are not intended to address the welfare of stray cats; however, we have interpreted their welfare impact in the Table 1 below.

#### 2.2.2. Attitudes towards Controlling Stray Cats

Understanding the relationship that people have with stray cats is important for understanding the perceived acceptability of management methods, which has implications for public support for legislation or regulations [26]. For starters, members of the public may be unaware of stray cats in their communities [27], which has implications for whether they are concerned about their welfare. Although stray cats may be considered a pest in some places in New Zealand, this does not mean that the public finds their lethal control acceptable [12]. Occupation, gender, and the degree of urbanisation where one lives were related to a higher acceptability of non-lethal methods for stray cats than for feral cats [26]. However, the differences in acceptability could in part be related to how lethal control is conducted. Half the survey respondents thought that stray cats should be assessed and euthanised (although who would do the euthanasia is not specified) [27], but this is categorically different to lethal management that includes traps, shooting, and poison. There may be decreasing concern about the humaneness of methods for controlling cats the more distant the relationship between human and cat; however, non-lethal control methods are still favoured more for stray cats than feral cats [12].

#### 2.2.3. Comparison of Laws and Regulations for Stray Cats

In all jurisdictions in Australia, the abandonment of cats is an offense under animal welfare legislation [28], and for Tasmania and Victoria, it is also an offense under animal management legislation [29]. Victoria prohibits feeding unowned cats under their Domestic Animals Act 1994 [30], whereas Queensland prohibits this practice under the Biosecurity Act 2014 [31]. The overwhelming majority of cats that enter Australian municipal animal facilities and shelters are stray [3,32,33]. Most stray cats are lethally managed through trap and kill, and practices, such as TNR, are not permitted in Australia [33,34].

There is little known about Australian attitudes towards urban stray cat management. Some people who provide care to cats do not consider themselves the cat’s owner (i.e., semi-owned cats), and maintain a balance of beliefs that they are helping and that the cats are independent [35]. A recent survey of Brisbane (Queensland) residents found that 79% of respondents preferred TNR to lethal management or no management of urban stray cats [33].

### 2.3. Regulating the Companion Cat’s Body: Limiting Reproduction

There are about 1.2 million companion cats in 41% of households across New Zealand [1]. Approximately 88% of New Zealand owners desex their cat [1], which is relatively high. However, there has been a downward trend from previous reports, where 93.2 and 93% of owners reported desexing their cats [27,36]. Although there is a relatively high rate of desexing of companion cats in New Zealand, the age at which these cats are desexed, and if they had a litter of kittens before desexing, is unknown and may impact upon metapopulation numbers.

#### 2.3.1. Welfare Benefits of Desexing and Prepubertal Desexing

There are many animal welfare-related benefits of desexing cats, including: reduced relinquishment to shelters and subsequent euthanasia [4,35,37,38]; decreased risk of reproductive disease, including cancers, infections, and tumours [38,39]; increased lifespan for cats [40]; reduced hyperactivity, aggression, and sexually motivated frustration, and increased affectionate behaviour [39,41,42,43]; and reduced risks associated with roaming, urine marking, and vocalizing [39,44].

Prepubertal desexing is typically performed between three and five months of age and is also referred to as ‘early-age desexing’ because it is performed earlier than the traditional six months of age [3,45,46]. The welfare benefits from prepubertal desexing include faster surgical procedures with less trauma and stress for the cat, fewer complications, and reduced recovery times [47]. Prepubertal desexing also benefits cat population management [48,49,50,51,52] by reducing the chance of litters born prior to desexing. Prepubertal desexing is a safe procedure and can be performed from 6 weeks of age [53], with no difference in health and behaviour outcomes for cats desexed under 12 weeks of age compared to those over 12 weeks of age [41,54].

#### 2.3.2. Laws and Regulations for Desexing

There are few places in New Zealand where desexing is required and only one of these examples can be considered prepubertal desexing. These examples are described in Table 2 below.

#### 2.3.3. Attitudes towards Desexing

While there are numerous benefits of desexing, including prepubertal desexing, for the individual cat, the mandating of these practices is aimed at population control, and specifically for reducing the number of unwanted cats and kittens [49,50]. Kittens in shelters are routinely desexed between six and eight weeks of age [59,60].

The majority of New Zealanders (64%) support mandatory desexing [27]. However, there are challenges with increasing the number of cats that are desexed New Zealand. The most common reason for not desexing cats is the cost and general feeling it is not necessary, and some owners believe it is beneficial for a cat to have a litter before desexing [1,27]. Many owners are unaware that their cat may reach puberty by four months of age, which is well before the traditional desexing age of six months [61]. Recent examples of where local regulations mandating desexing failed to pass or were purposely not included in the bylaw review include:

- In November 2021, Mackenzie District Council did not include a proposal for desexing and microchipping in their Keeping of Animals bylaws review. However, desexing and microchipping were recommended as responsible cat ownership behaviours. The final bylaw acknowledges that a lack of legislation for microchipping and registering cats limits their ability to mandate it through a bylaw. The bylaw further indicates that the recommendation to desex and microchip cats will be considered when an application is made for residents to obtain more than the two cats, as permitted under the bylaw. Even though desexing and microchipping are not explicitly required, these behaviours can still be factored into decision making regarding cats [62].
- In September 2020, Selwyn District Council removed mandatory desexing (and microchipping) in their public consultation for their new animal control bylaws, due to the behaviour being too difficult to enforce and the Council having no ability to issue fines or fund enforcement without national legislation [63].
- In 2019, Lower Hutt District Council had originally included mandatory desexing as part of the new proposed animal control bylaws. However, they decided to not seek a requirement for desexing cats. A survey of Lower Hutt residents indicated a ‘promote and educate’ approach was more favoured, and there was a lack of complaints related to cats [64]. Notably, 99% of survey respondents indicated that their cats were desexed, and over 53% of cat owners and 65% of non-cat owners supported mandatory desexing through bylaws [64].

While the benefits of desexing cats are well documented, the benefits of mandating desexing are less clear. A lack of evidence to support mandating desexing is in part due to poor data collection on what this type of regulation aims to achieve, such as a reduction in the number of animals entering shelters [65,66]. Mandatory desexing is intended to reduce the shelter intake of unwanted kittens from owned cats or the owner surrender of cats because of unwanted breeding [66]. However, most unwanted cats and kittens that end up in shelters do not have an owner [3,4]. Therefore, it is difficult to determine how mandating desexing would impact these cats.

The New Zealand Veterinary Association (NZVA) supports the prepubertal desexing of cats [67]. However, there is resistance to prepubertal desexing within the veterinary community [48,49] and concerns persist about the long-term health complications [61].

#### 2.3.4. Comparison with Laws and Regulations for Desexing

In the past 20 years, Australia has enacted mandatory desexing in four areas (Australian Capital Territory, South Australia, Tasmania, and Western Australia) [29]. The Australian Capital Territory (ACT) is the only municipality that has enacted prepubertal desexing where a kitten must be desexed by three months of age [68]. There is limited evaluation of the impacts of desexing legislation in Australia and there is little indication that it has achieved meaningful change in the shelter intake of unwanted cats and kittens and euthanasia [65].

A 2019 review of the Western Australia Cat Act 2011 found that 37% of respondents felt the age of 6 months was appropriate, 46% were unsure, and only 16% felt it was not appropriate [69]. Desexing rates for cats are relatively high in Australia; a recent study found that 83% of companion cats are desexed [70]. Previous studies have reported rates of desexing related to age, with 93% of cats over the age of two being desexed [46]. However, a recent study found that from 2010 to 2017, only 60% of cats had been desexed by 6 months, and 21.5% had been desexed by 4 months of age [70], indicating that there are many cats not desexed before puberty. A high number of well-socialised kittens from owned litters are surrendered to shelters [4,37], and although many may be from stray cats with carers, a proportion are likely from owned companion cats producing kittens before they are desexed [4]. In Australia, between 12 and 20% of cats have a litter before they are desexed [61].

A challenge with legislating the age at which cats are desexed is that this is highly reliant on veterinarians providing this service. Four to five months is considered the optimal age for desexing owned companion cats in Australia and New Zealand [61]. One particular concern is that legislating the age at which cats are desexed does not align with current veterinary practices [45]. A 2019 survey found that very few veterinarians in the ACT provided desexing at 3 months of age, even though this is the age at which cats must be desexed under the law [71]. Additionally, veterinary students in Australia and New Zealand are not commonly graduating with the knowledge and skills to perform prepubertal desexing [61]. The Australian Veterinary Association (AVA) supports desexing; however, it is not supportive of mandating the practice, nor the age at which to do this [64]. Prepubertal desexing is also supported by the American Veterinary Medical Association (AVMA) [72] and the British Veterinary Association (BVA) [73]. However, neither of these organisations supports mandatory desexing.

The failure to achieve improvements in targeted outcomes and, in some cases, worse outcomes, has occurred in the US, such as with increased owner relinquishment [65,66]. Where there is a decreasing, yet still high number of animals ending up in shelters, mandatory desexing of all or most animals is uncommon: only one US state has enforced the mandatory desexing of all cats (with some limitations), and some local jurisdictions require most cats to be desexed [74].

### 2.4. Regulating the Companion Cat’s Identity: Microchipping and Registration, Collars

Microchipping and microchip registration can help to ensure that a lost or injured cat’s owner can be identified and contacted. This can be especially true during emergencies. During the 2011 Christchurch earthquake, 85% of owners of microchipped animals were contacted within 3 h by the New Zealand Companion Animal Register, compared to only 25% of non-microchipped animals reunited with their owners within a 7-day period [75]. In areas where cats are targets of pest control, microchipping and microchip registration or other forms of identification can help to distinguish owned or managed stray cats from feral cats in pest management plans. There is no requirement to register cats with a government managed database at any level in New Zealand. However, some of the bylaws (noted below) mandate that registration must be performed on the New Zealand Companion Animal Registry.

#### 2.4.1. Laws and Regulations for Identification

Microchipping and microchip registration has been legislated at several different levels of government, and these are described in Table 3.

#### 2.4.2. Attitudes towards Identification

Microchipping and microchip registration is a well-supported management tool for cats in New Zealand, with 80% of the general public in favour of a national requirement for mandatory microchipping (in addition to a restriction of cat numbers and mandatory desexing) [15]. New Zealand benefits from having one database for registering companion animals (in contrast to Australia, which has six licensed databases), which likely reduces the challenges of aligning different systems [79]. The number of microchipped cats in New Zealand is increasing: 31.2% were reported microchipped in 2019 [27] while, more recently, in 2020, 49% were reported microchipped (with 36% reported registered) [1].

There is nuance in the extent to which people find it acceptable to use compulsory microchipping as a cat management tool. One study found that the majority of respondents indicated that they thought it was acceptable to use compulsory microchipping to distinguish owned cats from stray or feral cats in areas where there were high levels of biodiversity in Auckland; however, residents living near these areas were more likely to find this practice unacceptable than residents of other areas of Auckland [80]. Consistent with other studies demonstrating that cat owners are less accepting of management practices, cat owners found this use of microchipping less acceptable than non-owners [80].

Solely relying on microchipping as the only form of identification may limit the capacity to locate owners efficiently; microchips are not visible, require access to a microchip reader, and rely on the information linked with the microchip being accurate. A challenge for lost cats that enter shelters with a microchip is that their data associated with their microchip are inaccurate or not registered [1]; this makes reuniting cats with their owners difficult [3]. Collar use, however, does not appear to be a popular management technique, with studies reporting collars being worn by only approximately 1/3 of all owned cats in New Zealand (27.1%, [27]; 35.9%, [81]). Reasons for not using collars include cat intolerance of collars, repeated collar loss, and concern over collar safety [80].

#### 2.4.3. Comparison of Laws and Regulations for Identification

All states and the ACT legislate that cat owners must microchip their cats, with Queensland and Victoria requiring microchipping prior to sale or transfer of ownership, and the remaining jurisdictions requiring microchipping before a specific age. Western Australia requires that cats wear a collar with a visible identification tag [69]. Mandatory registration on government registries provides mechanisms, such as funding, for enforcing related bylaws.

Microchipping cats is supported as a responsible cat ownership behaviour, with one study indicating that over 95% of survey respondents (both cat owners and non-owners) agreed with the practice and over 76% of owners indicating that their cat was microchipped [82]. Sixty-five percent of survey respondents in Western Australia supported the requirement for cats to wear a council registration tag to help identify this cat as an owned companion animal and 60% of respondents agreed that microchips are effective at identifying cats [69]. With the potential for inaccurate information to be associated with a microchip [83], additional identification, such as a collar and tag, can facilitate cat identification [3,84]. The introduction of mandatory cat identification (microchipping) has been associated with an increase in the reclaim rates of cats in the US and the ACT (in combination with registration and annual licensing) [29,84].

### 2.5. Regulating the Companion Cat’s Space: How Many and Where

Regulating the physical locations cats can and cannot inhabit includes a range of practices, such as limiting the number of cats that can be kept on a private property, banning ownership, and restricting cats from roaming from their home or into certain areas. Limiting the number of cats permitted at a property is intended to reduce nuisance to communities by limiting related odours and cat fights. Limited cats on a property can also reduce the potential for an owner being over capacity to provide care for their cats, or, in extreme cases, reduce hoarding. Limiting the number of animals on a property has alleviated concerns related to keeping animals on increasingly smaller properties, and to reduce the nuisance to other members of the community [28].

Keeping cats from roaming reduces risks, such as disease transmission, injuries or death from road traffic accidents, dogs, or other people, or becoming lost or straying [85,86]. Conversely, cat containment may result in negative health and welfare issues for cats, e.g., obesity and stress, if managed insufficiently [87]. Cat owners should provide their contained cats with an appropriately enriched environment and diet to mitigate potential problems and promote the positive welfare of their cats [88]. There is the potential for an increase in owner surrender or abandonment due to inability or undesirability of keeping cats contained.

#### 2.5.1. Laws and Regulations Related to the Cat’s Physical Space

Laws and regulations related to cat spaces range from many that limit the numbers of cats at a residence to a few that prohibit cats, and these are presented in Table 4.

#### 2.5.2. Attitudes towards Regulating Cat’s Physical Space

Limiting the number of cats permitted on a property is the most common regulatory approach to cat management in New Zealand. The majority of New Zealanders (70%) support limits on the number of cats owned per household [15]. Differences between cat owners and non-owners in terms of support for regulations that restrict a cat’s physical space are well documented. Cat owners are consistently less accepting of strict cat management practices, such as either night-time or full confinement, when compared to desexing [15,80,91,92,93,94], vaccination [92], and limiting the number of cats a person can keep [80]. Woolley and Hartley (2019) found that almost double the proportion of non-owners compared to owners agreed that local governments should enforce stricter regulations on ownership and that cat-free zones were a good solution for cat predation [95]. There is some evidence that proximity to areas containing high ecological value influences cat owners’ views on their cats roaming. Bassett et al. (2020) found that people who lived closer to sensitive ecological areas were more likely to perceive the impacts of cats on wildlife as important than those who lived further from these areas and were more likely to consider the strictest forms of cat management as acceptable [80]. However, only 13% of these respondents reported keeping cats in at night and 10% reported keeping cats in during the daytime as best practice. Additionally, cat containment is often not supported by veterinarians in New Zealand [94]. A 2019 survey found that 7.8% of respondents kept their cats indoors [27], with a more recent survey reporting that 11% of owners keep their cats indoors [1].

#### 2.5.3. Comparison of Laws and Regulations of a Cat’s Physical Space

Bylaws exist at the local government level in Australia, with most limiting cat numbers at a residence [29]. Keeping cats fully contained inside the house or to a fully contained outdoor enclosure is more common in the US and is increasing in Australia [82]. The ACT has a 24 h containment regulation across sixteen suburbs [68]. In Queensland, at least nine councils require containment to an owner’s property, and this is related to requirements to minimise nuisance to neighbours [29]. Recent proposals for containment have occurred in South Australia and New South Wales [29]. In Victoria, roaming cats can be seized and impounded if not identified [30] and in some locations are prohibited, with at least one council enacting 24 h containment [29]. The Western Australia Cat Act 2011 allows local governments to prohibit cats from areas because of nuisances, including killing wildlife [69].

Compliance with regulations relating to the confinement of cats at night is poorly understood, although it has been reported to vary between 32 and 80% in Australia [86], making assessment of its effectiveness difficult. A survey of Western Australians found that 88% of respondents agreed that cats should be kept in at night [69]. Community acceptance for cat containment varies; some studies show broad support [82,86] and others a lack of support, or even opposition [20].

## 3. Discussion

### 3.1. Challenges with the Current System

Concerns exist about legislating cat owner behaviour include the enforceability or ‘lack of teeth’ of a law; regulations are not effective if they are not enforceable. Additionally, poor adherence to some regulations can lead to potential spill-over effect to other regulation adherence. Failure to engage with cat owners’ views on these topics can lead to the rejection of regulations or genuine inability to comply. Local authorities that do not manage cats have traditionally argued that the lack of complaints about cats demonstrates that the nuisance caused by cats does not warrant action. However, in a survey conducted by the Wellington City Council, 45% of respondents had been ‘bothered by cat behaviours, including digging and toileting in gardens and lawns, attacking and killing wildlife and other people’s pets, fighting, getting into rubbish, stealing property and producing unwanted kittens’ [76] (p. 4). In areas where complaints to local councils are low, complaints may be received by animal welfare organisations rather than local councils.

The lack of awareness of legislation is a potential barrier to improving cat management. Farnworth et al. (2010) found that fewer than half of survey respondents were aware of New Zealand legislation or differences between stray and feral cats [96]. There is some evidence that a proportion of the public considers local government or animal welfare organisations responsible for managing strays [27]. Some have argued that the inclusion of the term ‘stray’ in the Code of Welfare Companion Cats could potentially protect many cats that are neither companion nor feral [97]. However, the Code of Welfare Companion Cats only includes stray cats in one small section with no minimum standards for their care. There remains the challenge of a general lack of awareness of laws, regulations, and codes of welfare related to cats [97]. However, there is also a need for different policy mechanisms to protect stray cats that are not considered to have an owner or person in charge of them.

### 3.2. Better Understanding of the Impacts of Laws and Regulations Is Needed

There is a poor understanding of the impacts of current regulations that aim to manage free-roaming cat behaviours, such as restrictions on ownership or mandatory desexing [89]. The perverse outcomes of mandatory desexing are a major reason for opposition to this type of legislation [64,74,98]. The Australian Cat Action Plan (2018) includes the mandatory desexing of cats at point of sale or transfer of ownership as one strategy to reduce the abandonment of cats [99]. The key point to consider for advocacy of the mandatory desexing of cats is that it must accompany supporting measures, such as access to low cost desexing services [98]. When the barrier is the cost of the procedure, regulations, such as mandatory desexing, will be best implemented alongside mechanisms that facilitate access to affordable desexing [27]. It will also be important to consider how regulations impact other key stakeholders besides a cat’s owner. Veterinarians are an important link in communicating with cat owners and ensuring that owned kittens are desexed before reproductive maturity [37,100,101,102]. Ensuring that veterinarians are on board with early-age desexing is critical before regulating this behaviour. Encouraging veterinarians to perform prepubertal desexing and providing more opportunities for training will help ensure that they are comfortable delivering this service [48].

A further challenge with the regulatory approach in New Zealand is the limited understanding of the current distribution of different types of cats around the country. Therefore, there is no good way to anticipate how management would impact them. Cat density for all types of cats varies [103,104], and knowing these numbers is important for regulators/local government to know how many people and cats are impacted by the regulatory process [89].

### 3.3. Better Understanding of the Cat–Human Relationship Is Needed

Cat management decisions will benefit from a better understanding of people’s relationships with their companion cats. Much of the research to date on cat owners has been driven with conservation in mind and has contributed to a better understanding of psychosocial phenomena, such as attitudes, social norms, and beliefs about one’s ability to keep cats at home [94,105,106,107]. Other research has focused on evaluating the communication strategies aimed at motivating owners to keep their cats from roaming [86,105]. These types of studies have found that cat owners consistently underestimate the impact or the importance of the impact of roaming cats on native wildlife [27,91,94,95,106]; owners are more motivated to protect their cat than wildlife and trusted advisors, such as veterinarians, can be important as messengers about the benefits of keeping cats at home [94,106] and the need for segmenting target audiences for key communication messages [105].

The perceptions of risks associated with roaming impact whether an owner allows their cat to roam; however, these perceptions are context driven [92]. This indicates the need to avoid universal approaches to motivate owners to keep their cats at home and that the acceptability of policy to restrict roaming will likely be contingent on perceptions of local risks. Both Hall et al. (2016) and Foreman-Worsley et al. (2021) found that cat owners in New Zealand were more concerned about the impacts their cats had on wildlife than in other regions in the world, with at least one of these studies indicating that this was the major reason for keeping their cats indoors only [91,108]. The protection of wildlife is not the strongest motivating factor for New Zealand cat owners (and owners elsewhere) to keep their cats at home [94,106]. However, this should not be entirely overlooked as a potential influence on the acceptance of regulations.

There are gaps in our understanding of how regulations would be perceived as impacting the human–cat relationship, and ultimately compliance. In the case of strict measures, such as prohibiting cat ownership, community engagement will likely benefit the regulatory process. A proposal to prohibit cat ownership unless the cat was desexed and microchipped was rejected in 2018 by residents of Omaui, a different village in the same region as Stewart Island/Rakiura, where a similar prohibition was enacted. Residents claimed that there was poor public consultation, and the proposed rule was removed from the final plan. Southland Regional Council acknowledged that ‘their proposed domestic cat rules were a flashpoint that created community tensions and factions. In retrospect the viability of the proposal was maybe not tested sufficiently by ES (Environment Southland) …’ [109] (p. 10). A better understanding of the human–cat bond could include how owners understand their cat as a member of their family and their surrounding community and the implications this has on allowing them to roam. Recent work focusing on understanding the owner–cat bond has provided insights into improving cat care and understanding the benefits and limitations of cat ownership [110], whereas other research has stressed the need to understand what cats mean in society, especially how this informs legislation and regulation [111].

There is also a need to better understand the relationship that people have with other free-roaming cats, such as stray and feral cats. The current New Zealand regulatory environment heavily focuses on owned cats, as the owner can be held accountable, but free-roaming cats live as a metapopulation, and successful humane and effective management is contingent on this reality. That a stray cat can be considered a pest in one part of New Zealand and not others may contribute to a patchwork quilt approach to humane management for stray cats. Comprehensive cat management should include practices that recognise the diversity of relationships that stray cats have with the people who provide them care, as this can directly impact compliance with regulations [35,112,113].

### 3.4. A New Zealand National Cat Act

With no overarching legislation that addresses cat management in New Zealand, one of the biggest challenges is that different outcomes and policy mechanisms are used to achieve different goals. Somerfield (2019) has argued that these divergent goals, intended to protect biodiversity and cat owner interests, have resulted in cats ‘liv(ing) in an ambiguous legislative state’ [11] (p. 27). There have also been arguments that policy mechanisms, such as local bylaws, are being used outside of their intended use. However, in July 2017, Local Government New Zealand passed a remit (51% in favour) supporting lobbying the government, in part allowing territorial authorities regulatory power to regulate desexing, microchipping, and TNR, which would bring needed uniformity to cat management [11,16]. Finally, as noted in the case for the Selwyn district, having no national legislation for cat management means that local councils do not feel empowered to create bylaws, including mandating mechanisms, such as government registration, to create funding for the enforcement of these bylaws.

There are no current examples of comprehensive national cat legislation. However, it is instructive to consider examples from Tasmania and Western Australia that have cat specific legislation at the state level. In the state of Western Australia, The Cat Act 2011 enables local governments to enforce requirements for the identification, registration, and sterilisation of domestic cats [69]. Cats must be registered with local governments by the time they are 6 months old, which then helps track that they are microchipped and desexed [69]. A recent review of the legislation reported that it has been well accepted in communities since it was first enacted in 2013. However, problems remain with the age at which cats are sterilised being too late to avoid unwanted pregnancies, inconsistency with the number of cats allowed per residence, and the need to include confinement requirements so that cats are not allowed to roam [69]. Tasmania has enacted the Cat Management Act 2009, which has the purpose of promoting responsible ownership and the welfare of cats, the effective management of cats, and reducing the negative impacts of cats on the environment [114]. The Tasmanian Cat Management Plan 2017–2022 proposed a legislative framework that incorporated both the Cat Management Act 2009 and the Biosecurity Act to provide clarity of responsibilities [115]. This plan stipulates that the state government is responsible for rolling out the plan and for the administration of the legislation, with the local governments responsible for the enforcement of the relevant legislation where they are able, and it is considered necessary [115]. These examples provide insight into how different levels of government can hold responsibility for cat management that is locally relevant and enforceable.

National legislation to manage cats in New Zealand would provide a regulatory framework for humane and effective cat management by allowing local government to enact bylaws to manage the negative impacts that cats have on biodiversity and as a source of nuisance in communities, while also promoting the welfare of all cats (companion, stray, and feral). This process would benefit from enabling local councils to require registration so that cats can be tracked and to fund enforcement. Currently, the different policy mechanisms used to manage cats are targeted for much narrower purposes (e.g., protection of biodiversity), which do not fully capture the range of values attached to cats in New Zealand [11]. This can lead to attempts to control cats for conservation purposes that are untenable to the public because they are perceived as inhumane. Importantly, that all cats in New Zealand are managed humanely is a salient societal value. Therefore, the social license to do so must be grounded in the protection of cat welfare [17]. Additionally, national legislation can address cats as a metapopulation and more effectively minimise the negative impacts of cats. Focusing on managing only some categories of cats is less effective at achieving goals, e.g., the removal of stray cats near ecologically sensitive areas, and will be less effective at protecting wildlife if companion cats are permitted to freely roam in these areas.

## 4. Conclusions

There is good reason for the New Zealand government to take a stronger leadership role in cat management. One argument is that people with cats enjoy the benefits of companionship, but do not pay the true cost for letting their cat roam, especially if they are not desexed. A second argument is that there is a failure of government to protect public goods, such as cat welfare and native wildlife, to reduce the transmission of toxoplasmosis to native marine mammals and pastoral animals, and to control nuisance in communities. An effective way forward for improving the welfare of cats through regulations would be the enactment of a National Cat Act that aims to improve the welfare of cats and address other societal values, such as protecting biodiversity and reducing nuisance. These issues are interrelated, and a more consistent approach to managing these could achieve improvements for diverse groups in New Zealand.

## Figures and Tables

**Table 1:** Laws and regulations for controlling stray cats.

Topic	Welfare Impact	Requirement	Source	Type
Stray hold, find owner	(+) Reunite companion cats that have become lost; rehome stray cats that are socialised. Provide humane treatment for a stray cat if needed. (−) Stress related to confinement at shelter, risk of exposure to disease at the shelter.	An approved organisation (e.g., SPCA NZ) that takes custody of stray cat must take reasonable steps to find the owner, is allowed to take reasonable steps to prevent or mitigate any suffering, and must hold a stray cat for a minimum period of seven days before selling or rehoming the animal. This stray hold time can be avoided or reduced for a cat considered wild or unsocialised, and severely distressed due to stray hold, or if the cat is diseased and this may impact other animals in custody.	Animal Welfare Act, 1999, Part 7, Section 141 [10]	National
Prohibition of feeding stray cats.	(+) Reduce the stray cat population through decrease of resources; discourage congregation of cats that may lead to fighting, spread of disease, breeding. (−) Limit on providing for the nutritional needs of cats that may rely on being fed by humans.	Prohibits feeding or providing shelter to pest cats on public or private land without permission of the occupier. Pest cats are defined as those that are not microchipped where it is required, or not microchipped and registered, and are free-living, unowned, and unsocialised and have limited to no relationship with or dependence upon humans.	2019–2039 Greater Wellington Regional Pest Management Plan [23]	Regional
Prohibition of moving stray cats	(+) Reduce the stray cat population through decrease in moving them to different locations. Potentially reduce abandonment. Reduce stress related to capture, transport, and relocation. (−) Not clear.	Prohibits moving unowned cats to specific areas in the region to or near islands that are cat-free or are considered sensitive ecological areas. An unowned cat is defined as one without a microchip or other means of identification that is unregistered and is within any site the council declares as having sensitive ecological value and in a rural area.	2020–2030 Auckland Regional Pest Management Plan [24]	Regional
Prohibition of feeding stray (and companion) cats	(+) Reduce the stray cat population through decrease of resources; discourage congregation of cats that may lead to fighting, spread of disease, breeding. (−) Limit on providing for the nutritional needs of cats that may rely on being fed by humans.	Prohibit a person from feeding cats within the regions that contain a resident breeding or roosting population of any threatened native bird, reptile, or amphibian. This rule applies to any cat and is not specific to stray cats.	2020–2030 Auckland Regional Pest Management Plan [24]	Regional
Stray cats are legally considered a pest	(−) Subject to lethal control such as shooting, trapping, or poisoning.	Stray cats are defined as companion or domestic cats that have been lost or abandoned and may have their needs indirectly supplied by humans and live around human centres.	2017–2027 Northland Regional Pest Management Plan [25]	Regional
Reducing abandonment	(+) Reduces the number of companion cats that are abandoned or deserted who are reliant on humans to meet their needs. (+) Reduces the number of companion cats that become stray.	It is an offense to desert an animal without provisioning for the animal’s physical, health, and behavioural needs.	Animal Welfare Act, 1999, Part 1, Section 14 [10]	National
Reducing abandonment	(+) Reduces the number of companion cats that are abandoned or deserted who are reliant on humans to meet their needs. (+) Reduces the number of companion cats that become stray.	Prohibits the abandonment of any cat within the Auckland region.	2020–2030 Auckland Regional Pest Management Plan [24]	Regional

(+) indicates a welfare benefit; (−) indicates a welfare harm.

**Table 2:** Laws and regulations for desexing.

Topic	Welfare Impact	Requirement	Source	Type
Mandatory desexing	(+) Reduced number of unwanted cats and kittens, reduced nuisance. (−) Increased surrender at shelters and abandonment.	Mandates desexing for all cats over six months of age, born after the 1st of July 2018 (exemptions are in place for registered breeders).	Palmerston North City Council 2018 bylaws [55]	City
Mandatory desexing	(+) Reduced number of unwanted cats and kittens, reduced nuisance. (−) Increased surrender at shelters and abandonment.	Allows for the council to include terms and conditions requiring desexing of cats if a person seeks approval to keep more than three cats or kittens over the age of six months on their property.	New Plymouth City Council 2020 bylaws [56]	City
Mandatory desexing; prepubertal desexing	(+) Reduced number of unwanted cats and kittens, reduced nuisance. (−) Increased surrender at shelters and abandonment.	Requires any cat over four months of age be desexed unless for breeding purposes and be nationally registered; or the owner provides a certificate from a veterinarian indicating that desexing will adversely affect the cat’s health and/or welfare (veterinarians, SPCA, and cat boarding premises are exempt from this requirement).	Whanganui District Council 2020 bylaws [57]	Local District
Mandatory desexing	(+) Benefits from desexing; reduction in unwanted cats and kittens; reduction in predation of native wildlife. (−) Increased surrender at shelters and abandonment.	Requires residents on Stewart Island/ Rakiura to desex any cat that they keep, hold, enclose, or otherwise harbour as an exemption from a prohibition of having cats (except Bengal cats) on the island; this requirement extends to any Southland residents who keep, hold, or otherwise harbour a Bengal cat.	Southland 2019 Regional Pest Management Plan [58]	Regional

(+) indicates a welfare benefit; (−) indicates a welfare harm.

**Table 3:** Laws and regulations for identification.

Topic	Welfare Impact	Requirement	Source	Type
Microchip and registration	(+) Ensure owners can keep companion cats. (−) Increase in relinquishment.	Requires all cats over the age of 12 weeks to be microchipped and registered on the New Zealand Companion Animal Register.	2016 Greater Wellington City Council bylaw [76]	City
Microchip and registration	(+) Ensure owners can keep companion cats. (−) Increase in relinquishment.	Requires all cats over six months of age and born after 1st of July 2018 to be microchipped and registered on the New Zealand Companion Animal Register.	2018 Palmerston North City Council bylaw [55]	City
Microchip and registration	(+) Ensure owners can keep companion cats. (−) Increase in relinquishment.	Requires any cat over four months of age to be microchipped and registered with the New Zealand Companion Animal Register.	2020 Whanganui District Council bylaw [57]	Local District
Microchip and registration	(+) Ensure owners can keep companion cats. (−) Increase in relinquishment.	Requires every person who keeps a cat over the age of four months to microchip and register the cat with the New Zealand Companion Animal Register or other approved registry.	2021 Selwyn District Council bylaw [77]	Local District
Microchip and registration	(+) Ensure owners can keep companion cats. (−) Increase in relinquishment.	Required if cat owners possess, keep, hold, enclose, or otherwise harbour Bengal cats (no exceptions are made for living on or travelling to Stewart Island/Rakiura and other offshore islands).	2019 Southland Regional Pest Management Plan [58]	Region
Microchip and registration	(+) Ensure owners can keep companion cats. (−) Increase in relinquishment.	Required if cat owners keep, hold, enclose, or otherwise harbour in place any cat either in transit to or present on Stewart Island/Rakiura.	2019 Southland Regional Pest Management Plan [58]	Region
Collar and registration	(+) Ensure cats are not subject to pest control. (−) Increase in the number of cats lethally managed as pests.	Cats without a collar/harness or microchip that are found outside the Gisborne urban area, or to be of rural ownership, are defined as a feral cat and subject to pest management.	2016–2026 Gisborne Regional Pest Management Plan [78]	Region
Microchip and registration	(+) Ensure cats are not subject to pest control. (−) Increase in the number of cats lethally managed as pests.	Will be used to distinguish pest cats from non-pest cats and subject to regulations.	2019–2039 Greater Wellington Regional Pest Management Plan [23]	Region
Microchip or other method and registration	(+) Ensure cats are not subject to pest control. (−) Increase in the number of cats lethally managed as pests.	Will be used to distinguish an unowned cat from an owned cat and thus subject to regulations.	2020–2030 Auckland Regional Pest Management Plan [24]	Region

(+) indicates a welfare benefit; (−) indicates a welfare harm.

**Table 4:** Laws and regulations related to the cat’s physical space.

Topic	Welfare Impact	Requirement	Source	Type
Limits on number of cats per residence	(+) Ensure owners can provide adequate care for companion cats. (−) Increase in relinquishment or abandonment.	Limits the number of cats over a certain age that can be kept at a residence. Some allow for a permit for keeping more than the stated limit.	See [89] for a detailed table of bylaws	City or District
Ban on keeping a cat	(−) Increase in relinquishment or abandonment.	No cats shall be introduced or kept on any residential lots due to their potential to be predators of the long-tailed bat.	Environment Court of New Zealand [90]	City Area
Ban on keeping a cat	(−) Increase in relinquishment or abandonment.	Residents on Stewart Island/Rakiura cannot possess, keep, hold, enclose, or otherwise harbour Bengal cats.	2019 Southland Regional Pest Management Plan [58]	District
Prohibit cats from an area	(+) Ensure owners can keep companion cats out of areas where they may be managed as a pest. (−) Cat could be managed as a pest.	Any owner of a cat must ensure that their cat does not enter an intensively managed site, as defined in the plan.	2020–2030 Auckland Regional Pest Management Plan [24]	Region

(+) indicates a welfare benefit; (−) indicates a welfare harm.

## Data Availability

No new data were collected or analysed for this study.
